# COVID‐19, crisis, and emotional stress: A biocultural perspective of their impact on growth and development for the next generation

**DOI:** 10.1002/ajhb.23474

**Published:** 2020-07-16

**Authors:** Barry Bogin, Carlos Varea

**Affiliations:** ^1^ School of Sport, Exercise and Health Sciences Loughborough University Loughborough UK; ^2^ UCSD/Salk Center for Academic Research and Training in Anthropogeny (CARTA) University of California San Diego California USA; ^3^ Department of Biology, Faculty of Sciences Madrid Autonomous University Madrid Spain


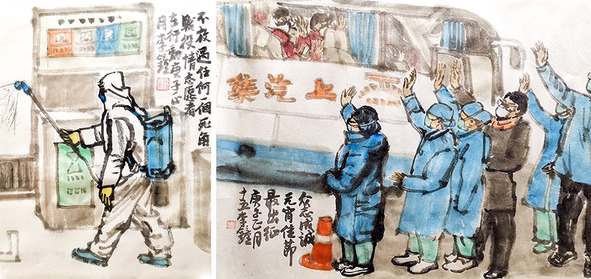


2020. «Paintings for Wuhan», Li Zhong (China) © Li Zhong through Tricontinental: Institute for Social Research. **Li Zhong** is president of the Artist Association of Fengxian District, Shanghai (China). An interview with Li Zhong and a series of paintings on the fight in China against the COVID‐19 pandemic area is available in Painting and epidemic.

## 1 HUMAN HEALTH MAY BE UNDERSTOOD BEST FROM A BIOCULTURAL PERSPECTIVE

This is a Commentary on the impact of the current COVID‐19 pandemic on future human growth and development. Our focus is on birth weight, one measure of prenatal growth and development, and its association with later life health. We assess the possibilities from the broad biocultural sense of human growth and development, especially in relation to its social‐economic‐political‐emotional (SEPE) correlates.

The biocultural perspective includes all aspects of human ecology in terms of biology, behavior, thinking, and beliefs. Human ecology and human biology are directly influenced by SEPE factors. A case in point is that on April 2, 2020 in the U.S. a freight train driver purposefully derailed his train to destroy the hospital ship *Mercy* sent to help with the COVID‐19 crisis. The train driver said he did it because he believed the *Mercy* was part of suspicious activities involving the coronavirus. In addition, during the first week of April, 5G cellular communication towers in Birmingham, Liverpool and Melling, UK were burned by people claiming that 5G technology is the cause of COVID‐19. Such emotionally and ideologically driven behavior always happen during pandemics. In the year 1630, a Bubonic Plague epidemic erupted in Milan, which also was at war with Spain. Four Spaniards in Milan were arrested, convicted of spreading Plague, and sentenced to death by torture. During the Plague pandemic of 1349 the people of Strasbourg, Alsace accused Jews of poisoning the water wells. One thousand Jews were arrested, taken to the cemetery, and burned alive. These cases and others of paranoia and murder during pandemics are described by medical historian Frank M Snowden in his book, *Epidemics and Society: From the Black Death to the Present* (Snowden, 2019).^1^

Fear of pandemic disease spreads as fast and as deep as the disease itself. The fear plays‐out in in many ways, from extremes of paranoia and violence, to xenophobia, closed borders, economic lockdowns, and social distancing. The fear pervades every level of society. The fear causes emotional stress. Chronic emotional stress—from insecurity that lasts for months or years—has biological impacts on people.

## 2 CRISIS, FEAR, AND BIRTH WEIGHT IN SPAIN

Along with several colleagues, we have been investigating the impact of chronic emotional stress on human growth. In one series of studies, we analyzed changes in birth weight related to the 2008 financial crisis in Spain. We reported a decline in birth weight across virtually all maternal social‐economic groups in Spain in the years leading up to, and especially, during the financial crisis (Terán, Juárez, Bernis, Bogin, & Varea, 2020; Terán, Varea, Juárez, Bernis, & Bogin, 2018; Varea, Terán, Bernis, Bogin, & González‐González, 2016). Our findings are supported by studies reporting reduced birth weight in Portugal, Iceland, Japan, and Greece during the 2008 banking system crisis, which was a global financial pandemic (Kana, Correia, Peleteiro, Severo, & Barros, 2017; Olafsson, 2016; Ueda, Kondo, & Fujiwara, 2015; Zografaki, Papamichail, & Panagiotopoulos, 2018).

Many factors influence human birth weight. A few well‐studied variables are maternal age, maternal size (height) and body composition, maternal psychological stress, gestational age at delivery, mother's hemoglobin concentration, and per capita daily income of the mother or family (reviewed in Bisai, Mahalanabis, & Bose, 2007; Bisai, Sen, Mahalanabis, Datta, & Bose, 2006). Other known influences of birth weight are maternal or fetal genetic anomalies; maternal exposure to infectious disease; maternal parasite load; maternal systemic diseases such as diabetes, hypertension, periodontal disease; abnormal placental development, and function that impairs fetal nutrition and oxygenation; maternal use or exposure to tobacco smoking, use of alcohol or many prescribed or illicit drugs (Negrato & Gomes, 2013). Maternal exposure to ambient air pollution is known to lower birth weight and increase the risk of preterm birth (Stieb, Chen, Eshoul, & Judek, 2012; Woodward, Finch, & Morgan, 2015).

While any or all these variables may influence birth weight, we interpreted the findings from our Spanish research in terms of SEPE factors. Our interpretation was guided by the fact that the entire Spanish population of births was similarly affected. This includes births to women of all household income levels, educational attainment, region of the country, urban or rural residence, body composition, health status, and exposure to air pollution. The Spanish National registry of births does not include most of these variables. However, given the population‐wide changes in birth weight, the impact of the 2008 financial crisis on pregnant women and birth outcome may have come about as an emotional response by mothers to the interaction of social, economic, and political factors at individual, family, and community levels. At all three levels, there was a reduction in material resources, such as jobs, money, and housing, deteriorating environmental conditions due to the closure or curtailment of public and private sector services in health, education, sanitation, and so forth, and a widespread increase in psycho‐social stress due to the insecurity of material resources, reliability of services, and uncertainty of the future. These SEPE sequelae of the 2008 financial crisis are tightly comingled, as the material, environmental, and psychosocial stress were caused by the political decisions of the Spanish government to impose harsh fiscal and social austerity policies on the entire population.

**FIGURE 1:**
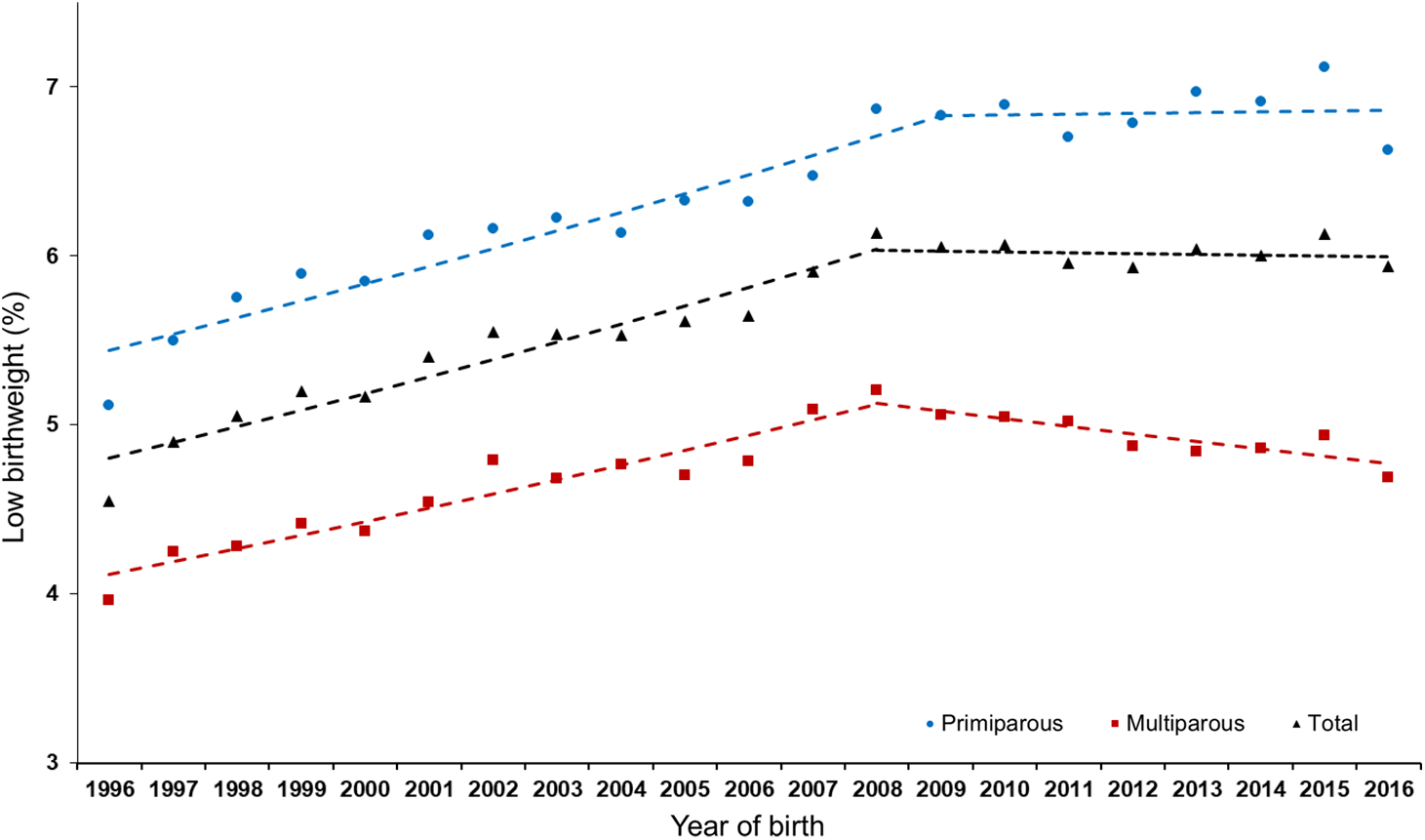
Trends in low birth weight prevalence by parity and total live single births, Spanish mothers, 1996 to 2016 (from Terán et al, 2020)

The uncertainties and psychosocial stress caused by the 2008 financial crisis and the governmental reactions to it could explain the Spanish birth weight reductions at two levels: first, at a population level, the crisis might determine changes in the sociodemographic profile of women who become mothers, with an increasing predominance of older women, who may have been at higher gestational or obstetrical risk; and second, at an individual level, the crisis might affect fetal development and birth outcome through direct worsening of living conditions and increased maternal stress during pregnancy. We analyzed the impact of both levels using a join point regression analysis to identify the time periods of significant changes in the prevalence of LBW by parity (Terán et al., 2020). Adjusted relative risk (RR) of LBW by year of birth was calculated in order to confirm that the time trend differences in LBW by parity were independent of possible confounders. The prevalence of LBW among singleton live births to primipara increased from 5.12% in 1996 to 6.87% in 2008 and then stabilized at that maximum value. Among live births to multipara LBW increased from 3.96% in 1996 to a maximum of 5.20% in 2008 and then significantly reduced (Figure 1). Trends in adjusted RR of LBW by parity confirm that primipara and multipara were affected differently by the financial crisis. Older, nulliparous women may have felt more biosocial pressure to reproduce during the financial crisis, compared with women who were already mothers. That biosocial pressure may have increased the risks for LBW. For all women, the global SEPE stress imposed in the years up to the financial crisis associated with a statistically and biologically significant increase in LBW.

## 3 MATERNAL STRESS AND LOW BIRTH WEIGHT

### 3.1 Contributions from the AJHB

**FIGURE 2:**
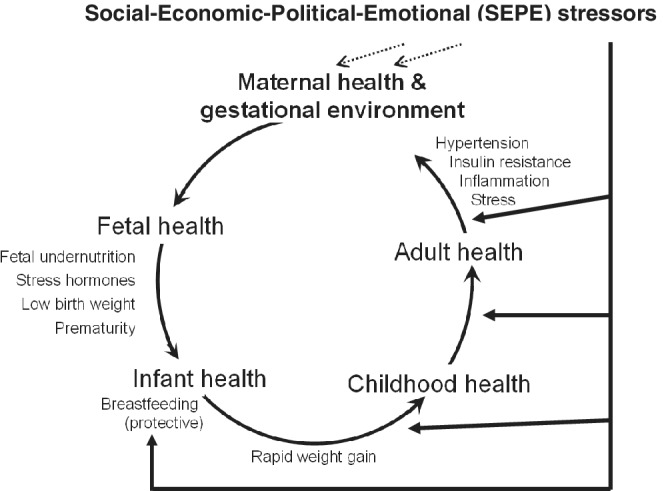
A life course, intergenerational model of Social‐Economic‐Political‐Emotional (SEPE) stressors and their influence on growth, development, and health. SEPE stressors may arise from government sanctioned or de factor racial/ethnic discrimination. Racism is a SEPE stressor that pervades virtually all biocultural levels of human existence—individual, family, and community. Racism and other SEPE stressors impact maternal biology and health, and the health of her offspring. Other SEPE influences may come from maternal behavior during pregnancy, such as diet and smoking, real or perceived insecurities with elevated production of stress hormones, and from decisions to formula feed or breastfeed and for how long. Maternal behavior and stress associated with SEPE factors shape neonatal health. Many neonatal health outcomes persist into adulthood, as they are maintained and further shaped by experiences of racism during growth and development. Poor adult health is not only a burden to the individual, but also influences the gestational and neonatal environment experienced across generations (original figure, based on Kuzawa, 2012)

There is a growing body of evidence in the literature for pathways from SEPE stress toward LBW. Several key articles were published in the *American Journal of Human Biology*. An evolutionary perspective on maternal stress and fetal responses was taken by Ivy Pike (2005). While she focused on preterm delivery, Pike did report that the stress of racial discrimination is associated with the consistently lower birth weights of African American neonates compared with European Americans. Countering claims that low birth weights for African Americans are due to genetic factors, Pike reviews the evidence published by David and Collins (1997) that recently immigrated women of African origin have higher birth weight babies than African Americans, but the risks for poor pregnancy outcomes increase with the duration of stay and exposure to racial discrimination of the immigrants in the United States. Discrimination and racism based on skin color, ethnicity, religion or any other characteristic of human groups is a powerful SEPE stress with measurable biological consequences. Building on Pike's review and analysis, Kuzawa and Sweet (2009) employed a social constructivist model to integrate the extensive literature showing how SEPE stresses become “embodied,” meaning in this case, how material and psychosocial discrimination are transduced into human biology, creating durable and transgenerational influences on health disparities in the United States (Figure 2). Kuzawa & Sweet's focus was on the social origin of prematurity and LBW in African Americans. They emphasized the biocultural pathways by which the effects of racial discrimination impact maternal stress physiology and lead to lower birth weight. In turn, lower birth weight babies are known to have higher risks for adult overweight and associated diseases. In this model, there are direct, “…links between early life environmental factors like maternal stress during pregnancy and adult race‐based health disparities in diseases like hypertension, diabetes, stroke, and coronary heart disease” (p. 2).

Empirical evidence for the transgenerational transmission of synergism between physiological stress and lower birth weight is provided in two studies by Zaneta Thayer and colleagues. They measured salivary cortisol, a widely used indicator of psychosocial and physiological stress. Both the absolute levels of salivary cortisol and its diurnal variation have been shown to be associated with reduced growth in body length (Nyberg et al., 2012). The first study (Thayer, Feranil, & Kuzawa, 2012), included a sample of 488 participants, mothers aged 20.8 to 22.3 years old, of the Cebu Longitudinal Health and Nutrition Survey, Philippines. Thayer and colleagues reported that mothers who were themselves born with lower birth weight had higher evening cortisol levels (*P* < .04) and that independently both lower maternal birth weight (*P* < .0001) and higher evening cortisol in adulthood (*P* = .003) predicted lower birth weight for male offspring. The associations for female offspring birth weight were not statistically significant (maternal birth weight, *P* = .07; bedtime cortisol, *P* = .3). The second study (Thayer & Kuzawa, 2014) was conducted in Auckland, New Zealand with a sample of 55 women at 34 to 36 weeks gestation. The socioeconomic status (SES) of the women was categorized using the locally validated NZ Deprivation Index for individuals (NZiDep). Maternal saliva samples were assessed for cortisol in the morning and at evening bedtime. Offspring salivary cortisol (N = 19) was assessed before and after a standard vaccination, a mild trauma, 6 weeks after birth. The authors reported that, “…after controlling for ethnicity and other covariates, women with higher NZiDep scores had significantly higher evening but similar morning cortisol, consistent with a pattern of chronic strain. Infants of women reporting greater material deprivation had elevated cortisol response to vaccination” (p. 723). Greater material deprivation and the chronic, daily stress this causes was associated with greater cortisol reactivity in both mothers and offspring. The authors review research showing that greater morning‐to‐evening reactivity and posttrauma reactivity are both predictive of poorer health now and in the future.

Research in Yucatan, Mexico connects the studies just reviewed, and many others on maternal SEPE stress and birth weight, to later life physical growth. Hugo Azcorra, Samantha Sanchez‐Escobedo and colleagues have been working with Maya families, all of whom are of low socioeconomic status (Azcorra, Dickinson, Bogin, Rodríguez, & Varela‐Silva, 2015; Sanchez‐Escobedo et al., 2020). One study included 109 families with measurements of a 6 to 8‐year‐old child, her/his mother, and the mother's mother (grandmother) living in Merida, the largest urban area of Yucatan. The researchers used total height and leg length as proxies for the quality of the material and SEPE environment. It was reported that maternal height and leg length were positively associated with child height and leg length. The association was relatively stronger for leg length, which is often a better index than total height of the quality of living conditions during infancy and childhood (Bogin & Varela‐Silva, 2010). Better living conditions of the grandmother in terms of house quality and larger family size were significantly associated with higher values of leg length in the grandchildren. All the grandmothers grew‐up in rural, agricultural areas where larger family size indicated more secure, less stressful living conditions. So, even in their current conditions of urban poverty, with daily insecurity from many material and SEPE stressors, early life conditions of rural‐living grandmothers were shown to have transgenerational effects on the linear growth of the grandchildren.

In another study the researchers worked with 260 dyads of Maya children aged 6 to 8 years and their mothers living in the city of Merida and the town of Motul, Yucatan, Mexico. The authors reported that child height‐for‐age was positively associated with the child's birth weight and with maternal height and mother's current age, but inversely associated with birth order and age of introduction of solid foods, that is, the age of termination of breastfeeding. These findings were interpreted to indicate that well‐being of the children, as measured by their height‐for‐age, is influenced by both the quality of SEPE living conditions when the mother was growing‐up and during her pregnancy, and by the child's own SEPE conditions. Taken together, these two Mexican studies and the others reviewed above, all support the life course, intergenerational model of SEPE stressors and their influence on birth weight and later growth, development, and health illustrated in Figure 2.

### 3.2 The larger landscape of stress and low birth weight

The SEPE stress and low birth weight research published in the *AJHB* is just the very top of the “tip of the iceberg” in terms all such publications. We mention here just a few more studies with some direct relevance to the COVID‐19 crisis and its probable effects on human growth and development.

A pathway from SEPE stressors to breastfeeding is shown in Figure 2. Kuzawa and Sweet (2009) reviewed evidence that breastfed infants grow slower, are less fat, and develop into adults with lower risks for hypertension, obesity, and diabetes. The associations between maternal anxiety during pregnancy, breastfeeding, and child growth were studied by Sinead English and colleagues (English, Wright, Ashburn, Ford, & Caramaschi, 2020). The researchers analyzed questionnaires and interviews collected as part of the Avon Longitudinal Study of Parents and Children, a birth cohort study of families from the Bristol region of the United Kingdom. The findings were that mothers reporting lower levels of anxiety were more likely to breastfeed, Odds Ratio (OR) = 1.17, and their breastfed infants grew more slowly before weaning. The same infants who were breastfed for more than 6 months had a later puberty onset in females, 1.5 months later than non‐breastfed infants after controlling for family social confounders and the girl's body mass index. Both slower infant growth in the first year and later age of puberty onset are, generally, beneficial for growth (greater adult height; lower fatness) and health. Breastfeeding did not influence puberty onset in boys, using age at voice change as the proxy for puberty.

Surveys in Los Angeles between the years 2007 and 2010 of 4970 women with singleton births reported that, “…financial life event stressors, but not other domains of stressors, were more likely to impact LBW among African Americans than Whites” (Zhao et al., 2015, p. 2195). The financial stressors faced disproportionately by African Americans, compared with European Americans, are, of course related to the racism, discrimination, and SEPE factors reviewed by Pike (2005) and Kuzawa and Sweet (2009). A *Nature* commentary by Harriet A. Washington titled, “How environmental racism fuels pandemics”, brings into sharp focus the connection between toxic SEPE living conditions and inflated COVID‐19 death rates for African Americans and other minority ethnic groups in the U.S., the UK, and elsewhere (Washington, 2020).

Disasters caused by weather, earthquakes, tsunamis, and other natural phenomenon or by war, terrorism, and other human activities are additional causes of stress. The September 11, 2001 terrorism events, culminating in the 9/11 destruction of the World Trade Center in New York City, exposed thousands of people to physical and emotional trauma with immediate and long‐term stress. One of the best studies, by Maslow and colleagues (Maslow, Caramanica, Li, Stellman, & Brackbill, 2016), examined reproductive outcomes (n = 3360) to pregnant women exposed directly to the events at the World Trade Center (WTC). Different types of exposure were identified and analyzed, including, “Having resided in the WTC area on 9/11…having been present in the cloud of dust that resulted from the collapse of buildings (dust cloud), having evacuated a building, having sustained 1 or more of several specified injuries, having been present in a building that collapsed, and having witnessed at least 3 of 5 specified potentially traumatizing events on 9/11…” (p. 1797). The women were assessed for posttraumatic stress disorder (PTSD) and birth outcomes in 2003 to 2004 (wave 1), and birth outcomes in 2007 to 2008 (wave 2), and 2011 to 2012 (wave 3). Maslow and colleagues observed that all types of direct exposure at the site and PTSD were associated independently with both LBW and preterm birth (PTB), but not with small‐for‐gestational age offspring. The researchers were able to show that exposure to particulate matter from the dust cloud had an independent contribution, not mediated by psychosocial stress, to both LBW and PTB. These adverse birth outcomes persisted for births up to 2 years after 9/11.

To summarize the studies reviewed here, and other research in the literature, we cite a systematic review and meta‐analysis of cohort studies that assessed the risk of low birth weight or preterm labor for women who experienced stress during pregnancy (Lima et al., 2018). The review identified, “…8 cohort studies with about 8,271 pregnant women and 1,081,151 children…” (p. 1) of appropriate quality and eligible for analysis. There was a statistically significant association between antenatal stress exposure and increasing rates of low birth weight (overall effect of metanalysis *P* = .003, OR = 1.68 [95% Confidential Interval (CI) 1.19, 2.38]). There was no statistically significant difference between nonexposed and exposed groups in terms of preterm labor (overall effect of metanalysis *P* = .09, OR = 1.98 [95% CI 0.91 to 4.31].

The fundamental message from this review is *maternal stress lowers birth weight*.

## 4 HOW STRESS GETS “UNDER THE SKIN”

In the Discussion of our Spanish financial crisis birth weight study we noted that it has been known for decades that birth outcome is associated as much with perceived stress as it is with objective stressful life events (Varea et al., 2016). The wide and immediate increase in LBW leading up to the 2008 financial crisis may be one expression of this psychological effect. The findings of Maslow et al. (2016) for the 9/11 WTC crisis add more evidence, in that women who only witnessed the events also had higher risk for LBW. Individual‐level studies document the association between birth outcomes and maternal psychological stress, ranging from chronic anxiety and depressive symptoms to acute stressors, determined by both pregnancy‐specific and general life event anxiety (reviewed in Niere et al., 2020; Varea et al., 2016). Potential pathways through which the experience of psychosocial stress by the mother may lead to negative perinatal outcomes have been proposed. Dysregulation of the maternal hypothalamic‐pituitary‐adrenal (HPA) axis is an often‐mentioned pathway (reviewed in Varea et al., 2016). The maternal dysregulation leads to an increased transfer of glucocorticoids from mother to fetus, resulting in lower birth weight and shorter gestational age at delivery.

The impact of maternal stress on birth outcomes may also operate indirectly on birth outcomes through increased negative health practices such as addictive behaviors, reduced antenatal care, and unhealthy or insufficient maternal nutrition. Varea and colleagues' review noted that placental corticotrophin‐releasing hormone (CRH) secretion is stimulated by the maternal pituitary‐adrenal stress hormones ACTH, beta‐endorphin, and cortisol. The prevalence of preterm deliveries is associated strongly with maternal stress through increased levels of maternal plasma concentrations of placental CRH, which is involved in the timing of parturition.

Results from several studies suggested that the impact of maternal stress on fetal growth is strongest in the last trimester of pregnancy, when the velocity of fetal growth in weight is greatest. It is likely that last trimester fetal weight growth velocity is affected less by genetic factors and more by maternal and intrauterine environments, especially by compromised uteroplacental perfusion and excessive fetal exposure to maternal glucocorticoids (Negrato & Gomes, 2013).

Postnatal stress also limits growth. Physical, nutritional, and emotional stresses of institutionalization on height growth were reviewed by Johnson and Gunnar (2011). The authors were especially interested in the causes of psychosocial short stature, a type of growth failure in height that cannot be explained by nutritional deficiency, physical abuse, or clinical disease. They reported that risk for psychosocial short stature was directly associated with abnormal levels of baseline cortisol, the hormonal response to chronic stress. The persistent emotional stress of the institutionalized children and their increased level of stress hormones, especially CRH led to a stimulation of somatostatin production. Somatostatin is a major inhibitor of growth hormone (GH). Consequently, the constant elevation of CRH would lead to a downregulation of GH and significant reductions in the production of insulin‐like growth factor 1 (IGF‐1) in the liver and other tissues. IGF‐1 is the major regulating hormone for growth in height. Taking all these findings into consideration, Johnson and Gunnar (2011) concluded that chronic exposure to social stress was a major determinant of short stature of institutionalized children and acted independently of other insults such as poor nutrition and health care.

The hormonal interplay between stress hormones and linear growth is not limited to children in institutionalized settings. Social stress factors such as competition, subordination, isolation, and deprivation are frequently occurring processes in human societies (Bogin, in press; Hermanussen, Bogin, & Scheffler, 2019; Mansukoski et al., 2020). Stress due to insecurities of employment, food, housing, health care, and education is also common to adults and their offspring, even in high income nations (Holley & Mason, 2019; Loopstra, 2018). These types of insecurities are often correlated, and this exacerbates the real and perceived psychosocial stress. It is well‐established that these stress factors have growth‐limiting impacts, as abundant research is available on SEPE gradients in height (Bogin, Scheffler, & Hermanussen, 2017; Niere et al., 2020).

In addition to the impacts of stress on the CRH ‐ GH ‐ IGF‐1 neuroendocrine cascade, research published since 2018 highlights another pathway. This is the pathway from the perception of psychosocial fear, to glutamate uptake by osteoblasts (cells that form new bone tissue), to surges in production of osteocalcin (OC), to suppression of the parasympathetic nervous system, to the acute stress response (ASR, Berger et al., 2019). The ASR is evolutionarily ancient and adaptive toward short‐term protection and survival to a variety of physical, social, and emotional threats. Osteocalcin, which seems critical to inducing the ASR, is a potent and versatile hormone. The only source of OC is from osteoblast cells, but this hormone has only minor effects on bone mineralization and density. Instead, the OC is secreted into the blood stream where it is reported to control several physiological processes in an endocrine manner, such as glucose homeostasis and exercise capacity, brain development, cognition, and male fertility (Moser & van der Eerden, 2019, p. 1).

Experimental evidence with rodents and clinical observations with human patients indicate that various types of physical and emotional stress cause a rapid rise in OC release into the blood stream and the initiation of the ASR, including the release of glucocorticoid hormones, such as cortisol, as well as increases in temperature, energy expenditure, heart rate, and respiration (Berger et al., 2019). The hypermetabolic state of catabolism during the ASR breaks‐down body cells to liberate amino acids, fatty acids, and glucose for a response to the stress. In the short‐term, the catabolism of the ASR may be beneficial for immune response, wound repair, “fight or flight” in response to threats, and, even, dieting for weight loss.

In the long‐term the consequences are harmful because a chronic stress response results in permanent loss of tissue and growth stunting (Arlt & Stewart, 2005; Christiansen et al., 2007; Matthews & Battezzati, 1993). Pregnant women, their fetuses, and young people of all ages will suffer if exposed to chronic, toxic emotional stress. Chronic toxic stress takes a toll on human health, including the physical growth of people, as much as do food shortages and infection. In addition to low birth weight, toxic emotional stress is associated with susceptibility to disease (Chrousos & Gold, 1998) and dysregulated gene expression (Slavich & Cole, 2013).


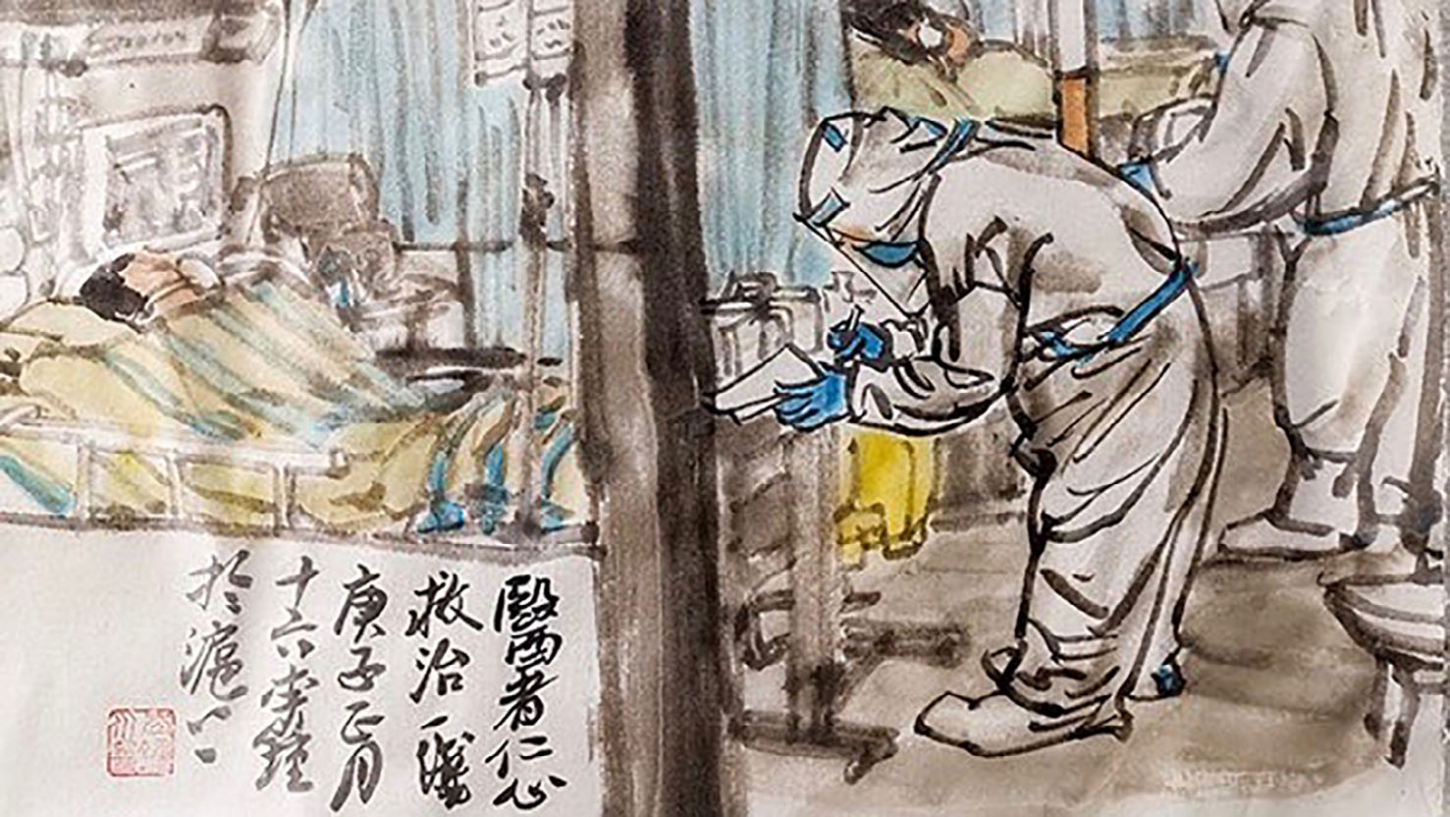


Li Zhong,《义务送药者》https://peoplesdispatch.org/2020/04/20/painting‐an‐epidemic‐an‐interview‐with‐li‐zhong/.

## 5 THE COVID‐19 CRISIS AFTERMATH

Birth weight is one of the key predictors of health risks at birth and later in life. The consequences of low birth weight include greater risk for infection, poor learning, and school performance, greater risk for psychological problems, reduced adult earnings, greater risk for adult overweight, diabetes, and heart disease, and, on average, an earlier age at death. Birth weight is under very strong stabilizing selection—not too low and not too high—and is therefore a key human biological characteristic and one that continues to drive human evolution. The current COVID‐19 crisis is biocultural in nature, with major and immediate SEPE impact. Economists for the European Union predict that “…the economy of the European Union is expected to shrink by 7.4 percent in 2020. By comparison, Europe's economy declined by just 4.4 percent in 2009, the worst year of the global financial crisis” (Birnbaum, 2020). The Chair of the US Federal Reserve Bank stated, “We are now experiencing a whole new level of uncertainty, as questions only the virus can answer complicate the outlook” (Smialek & Rappeport, 2020). Uncertainty creates fear and chronic fear is a toxic stress. It will take two or more generations to assess the biocultural consequences of the COVID‐19 crisis on people—from fetuses to the aged. One may hypothesize that for the immediate future there will be a global rise in maternal emotional stress and a decline in birth weight.

## AUTHOR CONTRIBUTIONS

**Barry Bogin:** Conceptualization; formal analysis; writing‐original draft. **Carlos Varea:** Conceptualization; writing‐review and editing.

## ENDNOTE

1 A recent interview with Frank M Snowden is available on Talk Radio Europe.
